# Anaerobic stress index to predict microbial ecological shifts in clear aligners

**DOI:** 10.1080/20002297.2026.2701561

**Published:** 2026-09-15

**Authors:** Mehmet Demirci, Ozge Unlu, Alpdogan Kantarci

**Affiliations:** a Faculty of Medicine, Department of Medical Microbiology, Kırklareli University, Kirklareli, Turkey; b Faculty of Medicine, Department of Medical Microbiology, Istanbul Atlas University, Istanbul, Turkey; c Department of Developmental and Surgical Sciences, University of Minnesota School of Dentistry, Minneapolis, MN, USA; d School of Dental Medicine, Harvard University, Boston, MA, USA

**Keywords:** Orthodontic dysbiosis, clear aligners, anaerobic stress index (ASI), silent hypoxia, oral microbiome, computational meta-analysis

## Abstract

**Objectives:**

Clear aligners are clinically perceived as periodontally ‘friendlier’ than fixed appliances (FA), yet localized gingivitis persists. Salivary diagnostics often mask site-specific dysbiosis through a ‘dilution effect’. This study aimed to characterize distinct ecological pressures and propose a novel anaerobic stress index (ASI) to quantify localized microbial burden.

**Methods:**

A computational meta-analysis was conducted using longitudinal 16S rRNA sequencing datasets (*n* = 976 samples from 10 distinct studies). Data was processed through a standardized Kraken2/Bracken pipeline. The ASI was calculated as the ratio of key anaerobic pathobionts (*Fusobacterium, Prevotella*) to facultative commensals (*Streptococcus, Rothia*).

**Results:**

Saliva acts as a ‘dilution filter,’ failing to capture sessile biofilm dynamics (AUC = 0.51 in FA). Fixed appliances exhibited mechanical dysbiosis with a 2.64-fold increase in *Actinomyces* (*p* < 0.0001) within the first month. Clear aligners induced a ‘silent hypoxia’. The supragingival biofilm showed a progressive shift toward anaerobiosis. The ASI in aligner patients quadrupled by month 6 (from 0.58 to 2.52; *p* = 0.002), a shift not detectable in matched saliva samples.

**Conclusions:**

Physical entrapment in fixed appliances versus micro-environmental hypoxia under clear aligners drives divergent dysbiotic pathways. The ASI serves as a robust biomarker for subclinical dysbiosis, suggesting periodic re-oxygenation therapeutics are necessary to restore host-microbe homeostasis.

## Article highlights

- Saliva testing often misses localized orthodontic dysbiosis due to a ‘dilution effect,’ underscoring the need for direct plaque sampling.
- Fixed appliances trigger mechanical dysbiosis with an early Actinomyces surge, while clear aligners create a stagnant microenvironment that leads to ‘silent hypoxia.’
- The Anaerobic Stress Index (ASI) directly measures this shift toward anaerobes, indicating that periodic re-oxygenation could be a useful clinical intervention.

## Introduction

Orthodontic intervention addresses both aesthetic demands and the need to restore occlusal function [1]. While fixed appliances have been considered the gold standard in orthodontic therapy, they are inherently associated with iatrogenic complications. Brackets and archwires create retentive sites for biofilm, impeding oral hygiene and frequently causing white spot lesions and gingival inflammation [2,3]. Clear aligners are popular, removable alternatives marketed for better aesthetics and easier oral hygiene [4]. Clear aligners were proposed to be ‘periodontally friendlier’ than fixed appliances, citing reduced plaque indices and lower gingival inflammation scores [2,5]. It was suggested that although clear aligners could induce dysbiosis, the resulting microbial shift was generally more compatible with oral health than that induced by fixed appliances [6]. While fixed appliances triggered cariogenic metabolic shifts and elevated specific periodontopathogens (e.g. *F. nucleatum, Actinomyces, Prevotella*), clear aligners appeared to preserve a much more stable microbial profile over 6 months [7,8]. On the other hand, white spot lesions and localised gingivitis associated with aligner patients suggested that distinct, perhaps overlooked, dysbiotic mechanisms were involved [9]. Targeted PCR-based analyses failed to detect broader ecological shifts [8,10]. Systematic reviews on orthodontic dysbiosis suggested that clear aligners support better periodontal health and a more stable microbiome than the pronounced periodontopathogenic shifts observed with fixed appliances [6,11], while aligners also trigger notable changes in biofilm composition [12]. The lack of consensus primarily stems from methodological variations across clinical trials, particularly inconsistent selection of sampling sites and a heavy reliance on non-invasive fluid diagnostics. Methodological heterogeneity, particularly the overreliance on non-invasive saliva sampling, further complicated any consensus on biofilm composition [13,14].

While studies using salivary 16S rRNA profiling indicated that the aligner microbiome was highly stable [15], the microbiome and metabolome of saliva and dental plaque differed significantly when exposed to appliances [7]. Acting as a transitional fluid, saliva pools microbes from across the oral cavity, inherently diluting the ‘ultra-local’ dysbiotic signals at the biofilm-enamel interface [16,17]. This ‘dilution effect’ is particularly critical in clear aligner therapy. Unlike fixed appliances, which accumulate biofilm around brackets, clear aligners cover the entire clinical crown and gingival margin for approximately 22 hours a day. This encapsulation restricts salivary flow and oxygen exchange at the tooth-biofilm interface, potentially creating a stagnant, hypoxic microenvironment [12]. Thus, relying solely on saliva samples could mask the localised ‘silent hypoxia,’ leading to an underestimation of the anaerobic stress imposed by aligners. Shifting from salivary surrogates to site-specific analysis of microbial dental biofilms is essential [13,18]. The advent of high-throughput 16S rRNA sequencing enables interrogation of these microbial shifts with unprecedented depth. Thus, we conducted a comprehensive computational meta-analysis of publicly available, longitudinal 16S rRNA datasets from the NCBI BioProject repository. This study integrated data from fixed-appliance and clear-aligner cohorts to achieve three objectives. First, we aimed to quantify the ‘dilution effect’ by showing that site-specific plaque sampling is essential for detecting orthodontic dysbiosis, whereas salivary diagnostics often fall short. Second, we evaluated how mechanical retention (fixed appliances) and environmental hypoxia (clear aligners) drive different ecological shifts. Based on these, we introduced and validated the Anaerobic Stress Index (ASI) as a metric to monitor localised hypoxic stress in these patients.

## Materials and methods

### Study design and data source

This study was conducted as a computational meta-analysis of publicly available high-throughput 16S rRNA amplicon sequencing data. The raw 16S rRNA sequencing data analysed in this study are publicly available in the NCBI BioProject repository under the accession numbers listed in **Supplementary Table S1**. The custom Python scripts used for statistical analysis, including the calculation of the anaerobic stress index (ASI) and ROC analysis, are available in the **Supplementary Material (File S1)** of this article.

A systematic query was performed in the NCBI BioProject and Sequence Read Archive (SRA) databases using the following search string: (microbiome OR microbiota OR metagenome) AND (orthodontic OR braces OR aligner OR fixed appliance OR denture). This initial search identified 41 candidate BioProjects. Projects were screened based on the following inclusion criteria: (1) longitudinal design involving human subjects; (2) availability of raw sequencing data (FASTQ) and complete metadata; and (3) comparison of fixed appliances (FA) or clear aligners (CA) against a matched control group. Projects were excluded if they lacked accessible raw data, contained corrupted sequencing files, presented metadata incompatibilities (e.g. inability to map sample IDs to FASTQ files or missing longitudinal time-point annotations), or were conducted on animal models. Following this rigorous filtration process, 10 distinct BioProjects met all quality control standards and were included in the final dataset. The control group consisted of systemically and periodontally healthy individuals who received no active orthodontic therapy and had no orthodontic treatment requirement during the observation period.

### Dataset characteristics and grouping

The final curated dataset comprised 976 samples retrieved from 10 distinct BioProjects. Samples were categorised based on the site of collection: Supragingival microbial biofilm (*n* = 652), saliva (*n* = 257), and gingival crevicular fluid (GCF, *n* = 67). To ensure temporal comparability across heterogeneous studies, longitudinal sampling time points were synchronised into four standardised intervals: baseline (T0, immediately before appliance bonding or insertion), followed by 1 month (T1), 3 months (T2), and 6 months (T3) of active treatment. Detailed characteristics of the included datasets are presented in Supplementary Table S1.

### Bioinformatics pipeline

Raw SRA FASTQ files were processed via the Galaxy platform following a standard pipeline (detailed in Supplementary material File S1): reads underwent quality control (FastQC/MultiQC), trimming (Trimmomatic), taxonomic classification (Kraken2), and abundance estimation (Bracken) before merging into a master dataset.

### Selection of microorganisms for the anaerobic stress index (ASI)

We selected taxa for the ASI based on well-established models of oral biofilm succession. Red complex pathogens (e.g. *Porphyromonas, Treponema*) were excluded because their very low abundance (<1%) in this largely non-periodontitis cohort would primarily introduce statistical noise. Instead, the ASI numerator comprised the orange complex bridge species *Fusobacterium* and *Prevotella*. As obligate anaerobes, they play a critical structural role in driving the transition from a healthy, oxygenated environment to an anaerobic subgingival state [19,20]. For the denominator, we selected *Streptococcus and Rothia*, which are typical early colonisers associated with healthy enamel surfaces. Finally, highly prevalent commensals such as *Veillonella* were omitted to preserve diagnostic specificity, as their abundance in both health and disease can mask early dysbiotic shifts.

### Calculation of the anaerobic stress index (ASI)

To quantify the hypoxic burden on the tooth surface, we used custom Python scripts to calculate the ASI. This index is defined as the ratio of the relative abundance of key bridge species (*Fusobacterium* and *Prevotella*) to health-associated facultative commensals (*Streptococcus* and *Rothia*):

$$\mathrm{Anaerobic}\;\mathrm{Stress}\;\mathrm{Index}\;(\mathrm{ASI})=\frac{\mathrm{Fusobacterium}+\mathrm{Prevotella}}{\mathrm{Streptococcus}+\mathrm{Rothia}}$$

This ratio captures the ecological shift from oxygen-rich, commensal environments to hypoxic, pathogen-rich niches by contrasting the relative abundance of *Fusobacterium and Prevotella*, well-established ‘bridge’ organisms facilitating anaerobic biofilm maturation [19,20], against *Streptococcus and Rothia*, the primary oxygen-tolerant colonisers of healthy enamel.

### Biomarker validation and matrix screening

To identify the most robust diagnostic window for orthodontic dysbiosis, we performed a comprehensive ‘matrix screening’ to evaluate metric stability by benchmarking the composite ASI against a simplified single-species ratio (*Fusobacterium*/*Streptococcus*), aiming to determine if a multi-marker index offers superior diagnostic resolution compared to tracking dominant antagonists alone. To establish site specificity, we compared the diagnostic accuracy of these metrics in saliva versus supragingival microbial biofilm, aiming to identify the most consistent sampling medium across both appliance cohorts.

### Statistical analysis

All downstream analyses and visualisations were executed using custom Python scripts that used the pandas, numpy, scipy, and scikit-learn libraries, as detailed in supplementary file S1. Given the non-normal distribution of the microbiome data, the Mann-Whitney U test was used to assess significant differences in bacterial abundance and to compare ASI scores across groups and time points. To evaluate the diagnostic utility of the proposed biomarker, receiver operating characteristic (ROC) curve analysis was conducted, with the area under the curve (AUC) calculated to quantify the index's ability to distinguish aligner-induced dysbiosis (T3) from baseline health (T0). Finally, data distribution and statistical results were visualised using the seaborn and matplotlib libraries, with statistical significance established at *p* < 0.05.

## Results

### General dataset characteristics and microbial diversity

**Figure 1.**
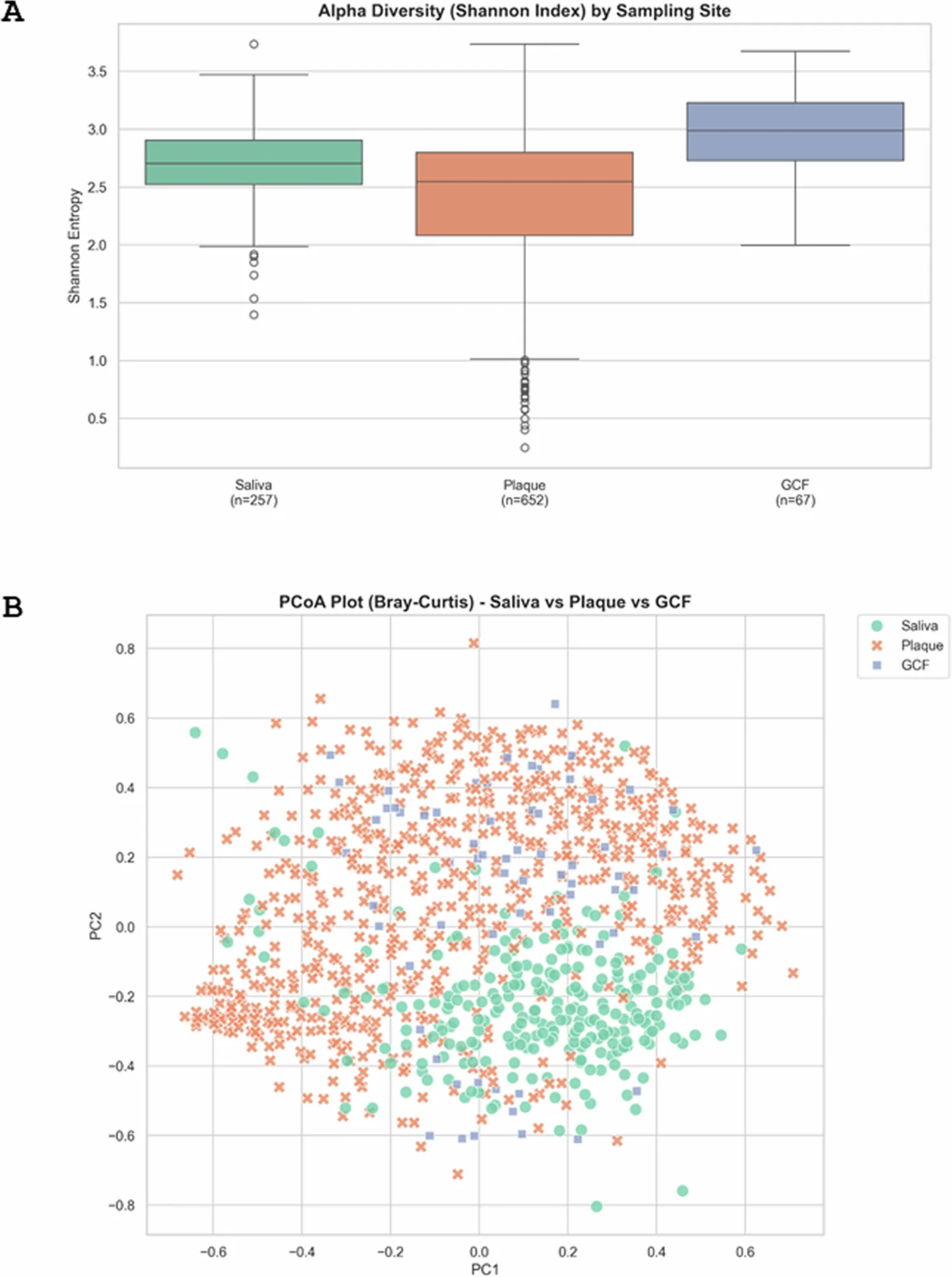
Shannon Index alpha diversity and a Principal Coordinate Analysis (PCoA) scatter plot showing distinct microbial clustering among plaque, saliva, and gingival crevicular fluid samples. A. Microbial diversity and compositional differences by sampling site. (A) Alpha Diversity (Shannon Index): Boxplots showing the distribution of diversity scores across gingival crevicular fluid (GCF), plaque, and saliva. Plaque (*n* = 652) exhibits significantly lower diversity compared to Saliva (*n* = 257) (Mann-Whitney U, *p* < 0.001), indicating a more specialised niche. B. Microbial diversity and compositional differences by sampling site. (B) Beta Diversity (PCoA): Principal Coordinate Analysis based on Bray-Curtis dissimilarity. Saliva samples (represented by a distinct colour) cluster separately from Plaque and GCF, confirming that saliva does not accurately reflect the biofilm community structure.

The final computational dataset comprised 976 samples (supragingival plaque: *n* = 652; saliva: *n* = 257; GCF: *n* = 67) obtained from 10 longitudinal studies. Alpha diversity analysis using the Shannon index revealed significant heterogeneity among sampling sites (*p* < 0.001). Contrary to the assumption that saliva reflects the localised biofilm environment, we observed distinct diversity profiles. GCF exhibited the highest diversity (mean Shannon index: 2.94 ± 0.37), followed by saliva (2.71 ± 0.33). Supragingival microbial biofilm, which represents the specific niche of orthodontic attachment, showed the lowest diversity (2.38 ± 0.62). Post-hoc Mann-Whitney U testing confirmed that saliva diversity was significantly different from microbial dental biofilm diversity (*p* < 0.001), suggesting that salivary metrics may overestimate the diversity of the localised biofilm (Figure 1A).

Beta-diversity analysis based on Bray-Curtis dissimilarity further corroborated these findings. Principal coordinate analysis (PCoA) revealed distinct clustering of samples by site, with saliva samples forming a tight, separate cluster distinct from the broader dispersion of microbial dental biofilm and GCF samples (Figure 1B).

### The ‘Dilution Effect’: taxonomic divergence between saliva, gingival crevicular fluid (GCF), and microbial dental biofilm

Genus-level data highlighted a clear ‘dilution effect,’ showing that salivary and GCF sampling capture communities distinct from the localised orthodontic biofilm. Saliva was enriched with taxa shed from mucosal surfaces (e.g. the tongue dorsum and buccal mucosa), effectively masking the site-specific signature of tooth-associated plaque. For example, *Prevotella* was much more abundant in saliva (13.28%) than in the dental biofilm (4.63%). In contrast, key biofilm organisms were largely diluted in fluid samples. *Actinomyces*, an early coloniser, accounted for 7.31% of the plaque community but was detected at much lower levels in saliva (1.31%) and at an intermediate abundance in GCF (8.10%). Similarly, *Corynebacterium,* an important structural component of plaque, was prominent in both plaque (5.26%) and GCF (6.77%) but nearly absent in saliva (0.70%). *Streptococcus*, the main early-colonising commensal, also showed higher abundance in plaque (22.97%) compared to saliva (14.04%) or GCF (8.74%). As shown in Table 1, these disparities indicate that fluid-based sampling captures a generalised oral microbiome, thereby diluting and obscuring the specific microbial shifts occurring at the gingival margin.

**Table 1.** Comparative relative abundance of key bacterial genera among saliva, gingival crevicular fluid (GCF), and supragingival plaque. This matrix highlights the ‘dilution effect,’ showing that salivary and GCF profiles significantly diverge from the localised, sessile biofilm community at the tooth-appliance interface. Data represent the mean relative abundance derived from the computational meta-analysis.

Genus	Ecological role	Saliva mean abundance (%)	GCF mean abundance (%)	Plaque mean abundance (%)	Fold difference
*Actinomyces*	Biofilm maturation & Bridging	1.31%	8.10%	7.31%	5.6x higher in plaque
*Corynebacterium*	Calculus formation & Structure	0.70%	6.77%	5.26%	7.5x higher in plaque
*Streptococcus*	Primary coloniser (Health)	14.04%	8.74%	22.97%	1.6x higher in plaque
*Prevotella*	Anaerobe/Shed bacterial noise	13.28%	0.00%	4.63%	2.9x higher in saliva
*Neisseria*	Early coloniser/Aerobe	11.30%	5.52%	5.54%	2.0x higher in saliva

**Figure 2.**
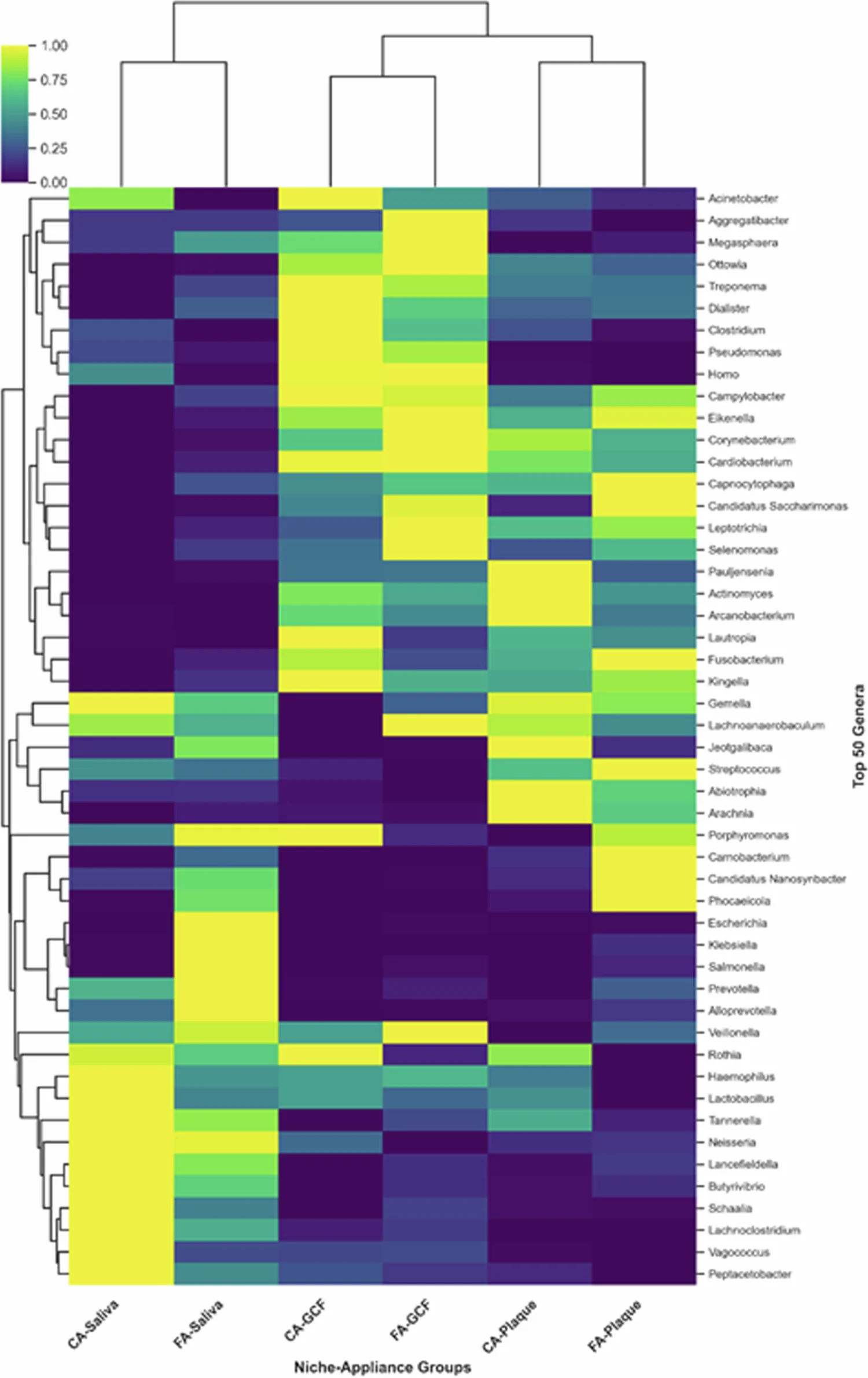
A dense hierarchical clustering heatmap with a dendrogram, using colour gradients to illustrate the divergent relative abundance of the top 50 bacterial genera across different orthodontic appliances and sampling sites. The comprehensive hierarchical clustering heatmap demonstrates the top 50 genera. A clustered heat map displaying the standardised relative abundance (Z-score) of the top 50 bacterial genera across fixed appliance (FA) and clear aligner (CA) cohorts. The vertical axis lists the genera, while the horizontal axis represents the study groups (Plaque, Saliva, GCF). Darker/brighter colours indicate higher relative abundance compared to the row mean. The clustering dendrogram (top) visually demonstrates the ‘dilution effect,’ showing that saliva samples cluster separately from site-specific plaque and GCF samples, regardless of appliance type.

A hierarchical clustering heatmap (Figure 2) confirmed niche-specificity across the top 50 abundant genera. This dense visualisation revealed a stark ‘ecological fingerprint’ for each sampling site. The dendrogram confirmed that saliva samples (FA-saliva, CA-saliva) formed a tight cluster, characterised by taxa shed from oral mucosal surfaces, and were distinctly separated from the sessile biofilm clusters of plaque. Notably, the plaque microbiome in fixed appliances and aligners shared a core compositional structure but exhibited subtle divergences in anaerobic density, which were visually resolved in the lower quadrants of the heatmap.

### Longitudinal shifts in fixed appliances: site-specific dysbiosis

**Figure 3.**
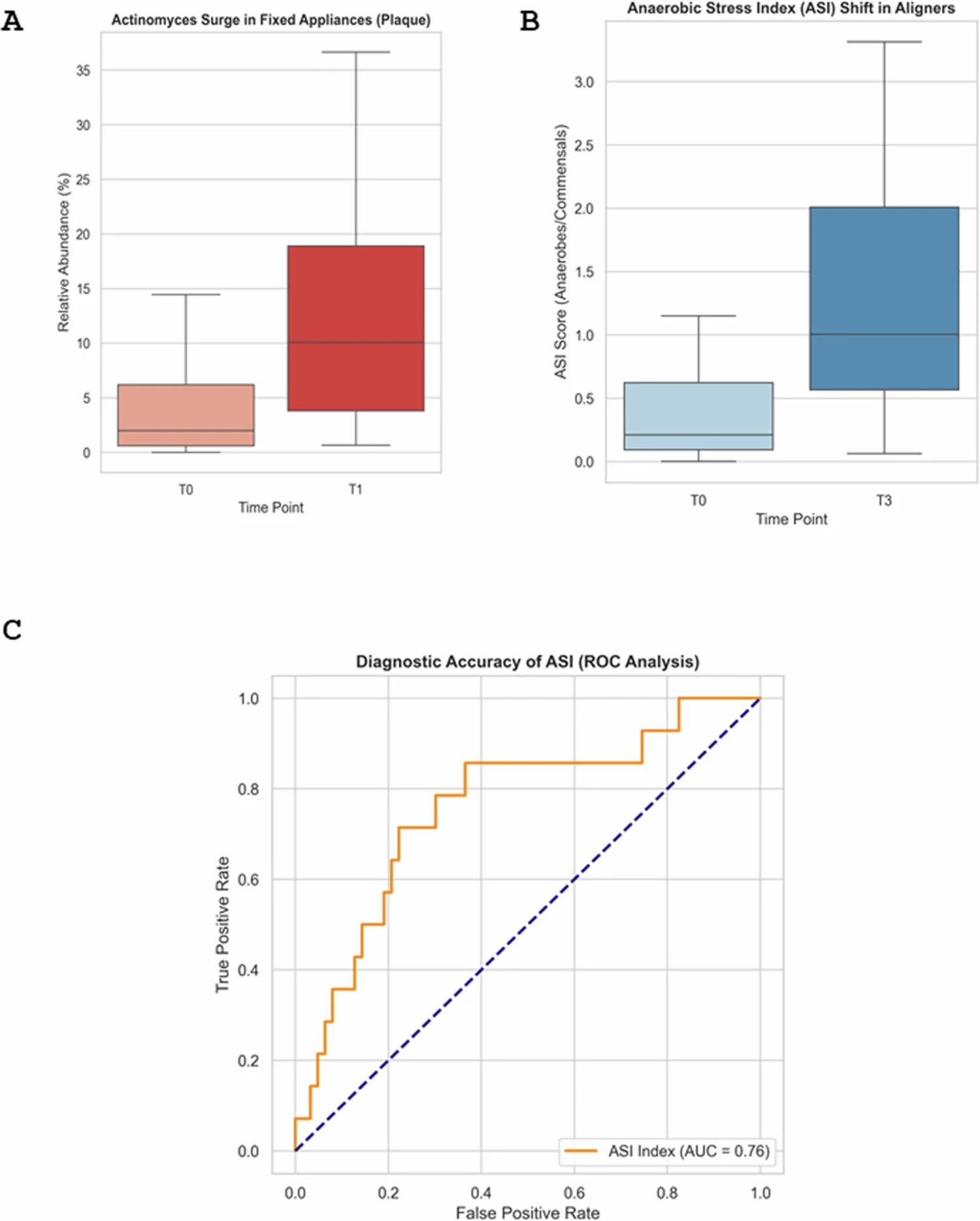
Longitudinal assessment of orthodontic-associated dysbiosis and the diagnostic performance of the Anaerobic Stress Index (ASI). (A) Actinomyces surge in fixed appliances: Longitudinal boxplot showing a 2.6-fold increase in relative abundance from baseline (T0) to 1 month (T1) (*p* < 0.0001). (B) Anaerobic shift in clear aligners: The ASI in plaque samples increased from 0.58 at baseline to 2.52 at 6 months (T3) (*p* = 0.002), quantifying the transition to a hypoxic environment (‘silent hypoxia’). (C) Diagnostic performance of the ASI: Receiver Operating Characteristic (ROC) curve demonstrating the ability of the ASI to discriminate between healthy baseline states and aligner-induced dysbiotic (hypoxic) states (AUC = 0.76).

Longitudinal analysis of the fixed appliance cohort revealed a discrepancy between sampling sites in detecting early dysbiotic shifts. Site-specific plaque analysis identified an acute ‘dysbiotic shock’ as early as the first month of treatment (T1). The relative abundance of *Actinomyces* in plaque surged significantly from a baseline mean of 5.01% at T0 to 13.25% at T1, representing a 2.64-fold increase (*p* < 0.0001; Figure 3A). This immediate proliferation of bridging organisms in plaque aligned with the clinical accumulation of biofilm around brackets, a phenomenon that saliva samples failed to capture with similar sensitivity. While GCF was characterised cross-sectionally due to its distinct compositional profile (Table 1), it was excluded from this longitudinal modelling because the available secondary datasets lacked the necessary paired temporal depth to construct a robust trajectory comparable to that of saliva and plaque.

### ‘Silent Hypoxia’ in clear aligners: The anaerobic stress index (ASI)

To evaluate the hypoxic burden imposed by clear aligners (CA), we applied the novel ASI, calculated as the ratio of key obligate anaerobes (*Fusobacterium, Prevotella*) to facultative commensals (*Streptococcus, Rothia*). Contrary to the presumed microbial stability, the plaque ASI increased from 0.58 at baseline to 2.52 at month 6 (a 4.31-fold increase; *p* = 0.002; Figure 3B), indicating a progressive anaerobic shift. To evaluate the diagnostic value of the ASI, we generated a Receiver Operating Characteristic (ROC) curve to classify samples as either pre-treatment (healthy) or aligner-treated (dysbiotic). With an Area Under the Curve (AUC) of 0.76 (Figure 3C), the ASI proved to be a reliable biomarker for detecting early-stage aligner-induced ecological stress, outperforming single-indicator species tracking.

To confirm that these longitudinal trends were not simply batch effects driven by a single outlier dataset, we examined the patterns within the individual BioProjects. The rise in *Actinomyces* during fixed appliance therapy and the increase in ASI with clear aligners were consistent across the independent studies. Furthermore, because the ASI relies on an internal ratio of taxa rather than raw relative abundances, it naturally accounts for cross-study variations. This confirms that the observed ‘silent hypoxia’ reflects a true biological response to orthodontic appliances, rather than an artefact of a specific study.

### Validation of sampling site selection

The rationale for prioritising plaque-based diagnostics over saliva was confirmed by our matrix screening analysis (Table 2). While the ASI proved to be a highly sensitive biomarker in clear aligner patients regardless of the sampling site (Saliva AUC = 0.90; Plaque AUC = 0.76), salivary diagnostics failed in the fixed appliance cohort. In fixed appliances, the salivary ASI yielded an AUC of 0.51 (equivalent to random chance), whereas the plaque-based ASI maintained diagnostic relevance (AUC = 0.78), statistically confirming the superior sensitivity of site-specific sampling over fluid-based diagnostics in capturing localised pathology.

**Table 2.** Diagnostic performance comparison (matrix screening) of the composite anaerobic stress index (ASI) versus a single-species ratio across different appliance types and sampling sites. AUC values range from 0.5 (random chance) to 1.0 (perfect discrimination). Although the simple ratio showed high sensitivity in some comparisons, the composite ASI was selected as the primary biomarker for its ecological robustness, incorporating multiple genus-level markers to minimise single-species volatility. Supragingival plaque was identified as the universal sampling site due to the consistent failure of salivary diagnostics in the fixed appliance cohort (AUC ≈ 0.50).

Appliance Type	Sampling Site	Tested Comparison	Simple Ratio AUC (Fuso/Strep)	Composite ASI AUC (Multi-marker Index)	Diagnostic Outcome
Clear Aligners	Saliva	T0 vs T3	0.83	0.90	Systemic spillover (High)
Clear Aligners	Plaque	T0 vs T3	0.82	0.76	Robust local signal
Fixed Appliances	Saliva	T0 vs T1/T3	0.40	0.51	Failed (Random chance)
Fixed Appliances	Plaque	T0 vs T1/T3	0.87	0.78	Robust local signal

## Discussion

Although clear aligner therapy is frequently associated with superior periodontal parameters compared to fixed appliances [5,6], the persistence of localised gingivitis and white spot lesions in clinical practice [9] presents a significant paradox. This discrepancy likely stems from methodological heterogeneity and reliance on salivary diagnostics [7]. We hypothesised that saliva acts as a transitional fluid that dilutes the ‘ultra-local’ signals of dysbiosis [16], thereby masking the stagnant, hypoxic microenvironment theoretically created by aligner encapsulation [12]. Consequently, this study employed a comprehensive ‘matrix screening’ to bypass these surrogate markers, aiming to characterise the precise ecological pressures, mechanical retention versus environmental hypoxia, driving orthodontic dysbiosis. Our analysis resolved this contradiction by exposing a ‘silent hypoxia’ phenomenon, a localised anaerobic shift that was statistically invisible in saliva but profoundly evident in the supragingival biofilm.

By critically evaluating saliva—the standard sampling medium in studies reporting minimal aligner dysbiosis [8,15]—our matrix screening reveals that this presumed stability is likely an inadvertent artefact of the ‘dilution effect’. This interpretation aligns with the broader literature consensus, which identifies saliva as a ‘poor surrogate’ for site-specific plaque communities due to high inter-study heterogeneity and conflicting diversity metrics [7,21]. We demonstrated that commensal taxa shed from oral mucosal surfaces, including *Prevotella* and *Neisseria,* dominate saliva. The high abundance of *Prevotella* in saliva likely reflects shedding from the dorsal tongue and mucosal surfaces rather than the tooth-associated biofilm, effectively masking the specific signals of sessile biofilm architects like *Actinomyces*. By relying on salivary proxies, prior studies likely underestimated the localised pathogenic burden. Thus, salivary sampling captures the shed bacterial flow from the oral mucosa but misses localised accumulation, akin to testing river water rather than the sediment bed [20]. Our matrix screening results reinforce this limitation, demonstrating that while the widespread dysbiosis in aligners may partially spill over into saliva, the localised hypoxic stress in fixed appliances is strictly confined to the biofilm-tooth interface. Consequently, these findings position supragingival plaque as a highly robust and representative medium for tracking appliance-specific dysbiotic shifts.

Regarding fixed appliances, our results align with those of Gong et al. [7], who identified *Actinomyces* as a key marker of dysbiosis. We observed a significant surge in bridging organisms within the first month of treatment. While Gong et al. linked general dysbiosis to appliance presence, our data suggested the *Actinomyces* surge likely stemmed from mechanical retention at the bracket-tooth interface. This rapid colonisation aligned with its established role as a core bridging organism, scaffolding secondary colonisers such as *Saccharibacteria* (TM7) [22]. Notably, this specific surge remained statistically undetectable in matched saliva samples, further validating the significant ‘dilution effect’ and reinforcing the need for plaque sampling to monitor localised bracket-induced risks [16].

In contrast to the mechanical retention observed in fixed appliances, clear aligners impose a distinct environmental selection pressure that has been largely overlooked in previous literature. While Rossini et al [11]. emphasised the ease of oral hygiene and ‘periodontal friendliness’ of removable appliances, this clinical perspective failed to account for the micro-environmental impact of the plastic barrier itself. By encapsulating the dentition, aligners restrict salivary flow and oxygen exchange, creating a stagnant microenvironment [12]. Dispelling the assumption that removable plastics are biologically inert, Zheng et al [23]. recently confirmed that aligners actively reduce plaque diversity and drive unique microbial signatures. Recent metagenomic data reinforced this ecological shift; within just three months, aligners upregulated adhesion-associated virulence genes (e.g. Type IV pili and flagella), indicating a strong selective pressure toward more pathogenic biofilm phenotypes [24]. ASI quantified this effect, revealing a quadrupling of the anaerobic burden, indicating rapid ecological succession in which oxygen-deprived niches selectively favour obligate anaerobes. Thus, the ASI may serve as a biological explanation for the ‘aligner paradox’, clarifying why teeth may appear macroscopically clean and exhibit reduced cariogenic loads [2], yet still harbour a biofilm shifting towards a pathogenic anaerobic profile. This phenomenon of ‘silent hypoxia’ could support the ecological plaque hypothesis proposed by Marsh [25] and further validated by Dong et al. [26], who demonstrated that significant metagenomic shifts occur in clinically non-cavitated sites (‘relative health’) long before lesions become visible, confirming that biological dysbiosis precedes macroscopic signs.

Current diagnostic frameworks lack a validated, orthodontic-specific index to monitor appliance-induced dysbiosis, as existing models such as the Red/Green (R/G) Ratio [27] are primarily tailored for generalised periodontitis. To bridge this gap, we propose the ASI as a targeted metric to quantify the ‘silent hypoxia’ phenomenon. This approach shifts the focus from merely counting bacteria to monitoring the ecological pressure. The necessity of such an ecological metric is empirically supported by Harzivartyan et al. [28], who demonstrated that standard hygiene protocols, ranging from mechanical brushing to chemical cleansing tablets, failed to achieve complete microbial eradication on aligner surfaces, highlighting the biofilm's resilience within this stagnant niche. Moreover, the significant increase in the ASI ratio suggests that aligner therapy requires targeted interventions to counteract hypoxia, such as the use of oxygenating mouthwashes or optimised wear protocols to allow periodic re-oxygenation, aiming to restore the host-microbe homoeostasis described in contemporary symbiotic models [29]. Beyond the oral cavity, this persistent, localised dysbiosis acts as a pathogenic reservoir that can drive systemic inflammation [30]. Ultimately, orthodontic guidelines should prioritise the ecological quality of the microenvironment over mere plaque quantity [25].

Relying on secondary data for this computational framework presents certain limitations. Because we aggregated data across multiple BioProjects, perfect intra-individual matching was not always feasible; as a result, our comparisons reflect cohort-level ecological trends rather than strictly patient-matched dynamics. The heterogeneity in the salivary datasets—particularly the mix of stimulated and unstimulated collection protocols—likely introduced variability, given how collection methods influence microbial profiles. Pooling datasets also brings inherent differences in sequencing platforms and targeted variable regions. The variance in our sampling timelines (e.g. T1 for fixed appliances vs. T3 for aligners) was largely driven by public data availability, though it also appropriately aligns with their distinct biological mechanisms: immediate mechanical retention versus gradual environmental hypoxia. Furthermore, a lack of detailed clinical metadata, such as periodontal pocket depths, prevented us from directly correlating microbial shifts with disease severity. We sought to minimise these confounding factors by using a ratio-based analysis. By depending on the internal proportions of antagonistic taxa rather than raw abundances, the ASI limits the impact of cross-study protocol differences. Ultimately, the large sample size (*n* = 976) provided the necessary statistical power to confirm that the observed ‘silent hypoxia’ is a genuine biological signal.

## Conclusion

This study demonstrated that while clear aligners may reduce the macroscopic quantity of plaque, they significantly alter its ecological quality through a mechanism of ‘silent hypoxia.’ Our computational meta-analysis confirmed that standard salivary diagnostics fail to capture this localised anaerobic shift due to a significant ‘dilution effect,’ necessitating site-specific plaque sampling for accurate risk assessment. Our findings delineate two divergent dysbiotic pathways: mechanical retention driving *Actinomyces* proliferation in fixed appliances, versus environmental selection cultivating pathogenic anaerobes under the hypoxic canopy of clear aligners. To address the diagnostic gap in monitoring this invisible risk, we propose the ASI as a novel approach, suggesting a paradigm shift from exclusively mechanical hygiene protocols toward a precision medicine framework. Integrating the ASI with clinical observation and computational modelling would allow orthodontists to bypass the limitations of generic salivary screening. This multidimensional approach reveals that future orthodontic care must prioritise targeted ecological therapeutics, such as periodic re-oxygenation, to counteract localised hypoxic stress.

## Supplementary Material

Supplementary Material Supplementary Table S1.docx

Supplementary Material FileS1.docx

## Data Availability

The raw 16S rRNA sequencing data analysed in this study are publicly available in the NCBI BioProject repository under the accession numbers listed in Supplementary **Table S1**. The custom Python scripts used for statistical analysis, including the calculation of the anaerobic stress index (ASI) and ROC analysis, are available in the **Supplementary Material (File S1)** of this article.
