# Supplementary material for: Anaerobic stress index to predict microbial ecological shifts in clear aligners
Source: J Oral Microbiol. 2026 Sep 15;18(1):2701561. doi: 10.1080/20002297.2026.2701561 (PMC13580376; doi:10.1080/20002297.2026.2701561)
Supplement: Supplementary file 1 [file ZJOM_A_2701561_SM5530.docx]

**Supplementary Material:**

**Supplementary File S1**

**Title: Anaerobic Stress Index to Predict Microbial Ecological Shifts in Clear Aligners**

This document contains the computational workflow and custom Python scripts used to process, analyze, and visualize the microbiome data presented in the main manuscript. It includes the statistical validation of the "Dilution Effect," the longitudinal tracking of the "T1 Shock," and the comprehensive "Matrix Screening" used to validate the Anaerobic Stress Index (ASI).

**1. Data Processing Workflow (Galaxy Platform)**

Raw FASTQ files were retrieved from the NCBI Sequence Read Archive (SRA). Data pre-processing was performed using the Galaxy platform (usegalaxy.org) with the following standard pipeline:

1. **Quality Control:** *FastQC* and *MultiQC* were used to assess read quality.
2. **Trimming:** *Trimmomatic* was used to remove low-quality bases and adapters (Sliding window: 4:20, Min length: 50).
3. **Taxonomic Classification:** *Kraken2* was used for assigning taxonomic labels to sequencing reads using the Standard Database.
4. **Abundance Estimation:** *Bracken* (Bayesian Reestimation of Abundance with KrakEN) was used to estimate the relative abundance of species/genera from Kraken2 reports. The resulting abundance tables were merged into a single master dataset (orthodontic_microbiome_data.xlsx) for downstream Python analysis.

### 2. Custom Python Scripts

**Prerequisites:** The following Python libraries are required: pandas, numpy, scipy, sklearn, matplotlib, seaborn.

#### Script A: Quantifying the "Dilution Effect" (Saliva vs. Plaque)

This script performs Mann-Whitney U tests to statistically validate the taxonomic divergence between saliva and plaque, confirming saliva as a "poor surrogate."

import pandas as pd

from scipy.stats import mannwhitneyu

\# CONFIGURATION

FILE_PATH = 'orthodontic_microbiome_data.xlsx' # Place your dataset in the working directory

def analyze_dilution_effect():

\# Load Data

try:

df = pd.read_excel(FILE_PATH, sheet_name='Genus_Relative_Abundance')

meta = pd.read_excel(FILE_PATH, sheet_name='Metadata')

except FileNotFoundError:

print("Dataset not found.")

return

\# Merge Data

merged = df.merge(meta, on='SampleID')

\# Define Comparison Groups

plaque = merged[merged['SampleType'] == 'Plaque']

saliva = merged[merged['SampleType'] == 'Saliva']

print("--- DILUTION EFFECT ANALYSIS (Mann-Whitney U) ---")

\# Key Taxa to Check

target_genera = ['Actinomyces', 'Streptococcus', 'Prevotella', 'Neisseria']

for genus in target_genera:

p_mean = plaque[genus].mean()

s_mean = saliva[genus].mean()

stat, p_value = mannwhitneyu(plaque[genus], saliva[genus])

print(f"\nGenus: {genus}")

print(f"Mean Abundance -> Plaque: {p_mean:.2f}%, Saliva: {s_mean:.2f}%")

print(f"P-value: {p_value:.5e} {'(SIGNIFICANT)' if p_value < 0.05 else ''}")

if __name__ == "__main__":

analyze_dilution_effect()

#### Script B: Longitudinal Dysbiosis Tracking (The "T1 Shock")

This script validates the specific surge of *Actinomyces* in Fixed Appliances during the first month (T0 vs T1).

import pandas as pd

from scipy.stats import mannwhitneyu

def analyze_t1_shock():

\# Load Data (Assuming same structure as above)

df = pd.read_excel('orthodontic_microbiome_data.xlsx', sheet_name='Merged_Data')

\# Filter: Fixed Appliances, Plaque Only

subset = df[(df['Group'] == 'Fixed_Appliance') & (df['SampleType'] == 'Plaque')]

print("\n--- T1 SHOCK ANALYSIS (Fixed Appliances) ---")

\# Compare T0 (Baseline) vs T1 (1 Month)

t0_data = subset[subset['TimePoint'] == 'T0']['Actinomyces']

t1_data = subset[subset['TimePoint'] == 'T1']['Actinomyces']

stat, p = mannwhitneyu(t0_data, t1_data, alternative='less') # Hypothesis: T0 < T1

print(f"Actinomyces Mean T0: {t0_data.mean():.2f}%")

print(f"Actinomyces Mean T1: {t1_data.mean():.2f}%")

print(f"Fold Increase: {t1_data.mean() / t0_data.mean():.2f}x")

print(f"Significance (P-value): {p:.5e}")

if __name__ == "__main__":

analyze_t1_shock()

#### Script C: "Matrix Screening" and ASI Validation

This is the core diagnostic script. It calculates the **ASI**, compares it against a **Single-Species Ratio**, and runs **ROC Analysis** to determine diagnostic accuracy (AUC) across different sampling sites.

import pandas as pd

import numpy as np

from sklearn.metrics import roc_auc_score

def calculate_ratios(row):

\# Epsilon to prevent division by zero

eps = 0.001

\# 1. Anaerobic Stress Index (ASI) - The Proposed Metric

\# Formula: (Fusobacterium + Prevotella) / (Streptococcus + Rothia)

asi = (row['Fusobacterium'] + row['Prevotella']) / (row['Streptococcus'] + row['Rothia'] + eps)

\# 2. Single Species Ratio (Benchmark)

\# Formula: Fusobacterium / Streptococcus

single = row['Fusobacterium'] / (row['Streptococcus'] + eps)

return pd.Series([asi, single], index=['ASI', 'Single_Ratio'])

def matrix_screening():

df = pd.read_excel('orthodontic_microbiome_data.xlsx', sheet_name='Merged_Data')

\# Apply Ratio Calculations

df[['ASI', 'Single_Ratio']] = df.apply(calculate_ratios, axis=1)

print("\n--- MATRIX SCREENING: DIAGNOSTIC ACCURACY (AUC) ---")

print("Task: Distinguishing Baseline (T0) from Dysbiosis (T3) in Clear Aligners")

results = []

\# Loop through Sites and Metrics

for site in ['Saliva', 'Plaque']:

subset = df[(df['Group'] == 'Clear_Aligner') & (df['SampleType'] == site)]

subset = subset[subset['TimePoint'].isin(['T0', 'T3'])]

\# Encode Target: T0=0 (Health), T3=1 (Dysbiosis)

y_true = subset['TimePoint'].apply(lambda x: 1 if x == 'T3' else 0)

\# Calculate AUC for ASI

auc_asi = roc_auc_score(y_true, subset['ASI'])

\# Calculate AUC for Single Ratio

auc_single = roc_auc_score(y_true, subset['Single_Ratio'])

results.append({'Site': site, 'ASI_AUC': auc_asi, 'Single_AUC': auc_single})

\# Output Table

res_df = pd.DataFrame(results)

print(res_df)

if __name__ == "__main__":

matrix_screening()
