# Supplementary material for: Anaerobic stress index to predict microbial ecological shifts in clear aligners
Source: J Oral Microbiol. 2026 Sep 15;18(1):2701561. doi: 10.1080/20002297.2026.2701561 (PMC13580376; doi:10.1080/20002297.2026.2701561)
Supplement: Supplementary file 2 [file ZJOM_A_2701561_SM5531.docx]

**Supplementary Table S1.** Characteristics of the included NCBI BioProjects and distribution of samples (n=976) across appliance types and sampling sites. *FA = Fixed Appliances, CA = Clear Aligners

| ID | Appliances* | Samples | Sequencing Platform | Location | Sample count (n) |
| --- | --- | --- | --- | --- | --- |
| PRJDB10426 | FA | Plaque, Saliva | Illumina MiSeq | Japan | 126 |
| PRJNA1064555 | FA, CA, Control | Plaque | PacBio Sequel | China | 48 |
| PRJNA1064902 | FA | Plaque | Illumina MiSeq | China | 54 |
| PRJNA1129082 | FA, CA | Plaque, Saliva | Illumina NovaSeq | China | 100 |
| PRJNA1225015 | FA | Plaque, Saliva | Illumina MiSeq | China | 64 |
| PRJNA1355306 | FA, CA | GCF, Plaque, Saliva | Illumina NovaSeq | China | 200 |
| PRJNA552608 | FA | Plaque | Illumina MiSeq | USA | 192 |
| PRJNA648248 | FA | Saliva | Illumina HiSeq | China | 57 |
| PRJNA895208 | FA | Plaque | Illumina MiSeq | China | 60 |
| PRJNA905724 | FA, CA | Plaque | Ion Torrent S5 | Australia | 75 |
| Total |  |  |  |  | **976** |
